# Endothelial cell activation and glycocalyx shedding - potential as biomarkers in patients with lupus nephritis

**DOI:** 10.3389/fimmu.2023.1251876

**Published:** 2023-10-03

**Authors:** Susan Yung, Tak Mao Chan

**Affiliations:** Department of Medicine, School of Clinical Medicine, The University of Hong Kong, Hong Kong, Hong Kong SAR, China

**Keywords:** lupus nephritis, glycocalyx, syndecan-1, thrombomodulin, hyaluronan, cell adhesion molecules

## Abstract

Lupus nephritis (LN) is a common and severe manifestation of systemic lupus erythematosus and an important cause of acute and chronic kidney injury. Early diagnosis of LN and preventing relapses are key to preserving renal reserve. However, due to the complexity and heterogeneity of the disease, clinical management remains challenging. Kidney biopsy remains the gold standard for confirming the diagnosis of LN and subsequent assessment of kidney histopathology, but it is invasive and cannot be repeated frequently. Current clinical indicators of kidney function such as proteinuria and serum creatinine level are non-specific and do not accurately reflect histopathological changes, while anti-dsDNA antibody and C3 levels reflect immunological status but not kidney injury. Identification of novel and specific biomarkers for LN is prerequisite to improve management. Renal function deterioration is associated with changes in the endothelial glycocalyx, a delicate gel-like layer located at the interface between the endothelium and bloodstream. Inflammation induces endothelial cell activation and shedding of glycocalyx constituents into the circulation. This review discusses the potential role of soluble glycocalyx components as biomarkers of active LN, especially in patients in whom conventional serological and biochemical markers do not appear helpful.

## 1 Introduction

Systemic lupus erythematosus (SLE) is an autoimmune disease characterized by a breakdown of immune tolerance, autoantibody production and immune-mediated injury to multiple organs. SLE has a wide spectrum of clinical manifestations. Kidney involvement, termed lupus nephritis (LN), affects up to 60% of patients, and is an important cause of morbidity and mortality (1). Improvement in immunosuppressive treatment over the past two decades has improved short-term clinical outcomes. Cardiovascular disease (CVD) has emerged as an important cause of morbidity and mortality during long-term follow-up (2). Lupus patients have increased risk of developing CVD compared to age- and gender-matched controls, and the risk ratio is even greater in premenopausal women with LN compared with age-matched controls (3–5). Increased prevalence of sub-clinical atherosclerosis has also been reported, with up to 40% of patients showing evidence of carotid plaque or increased coronary calcification (6, 7). Factors that contribute to cardiovascular events in lupus patients include not only the traditional Framingham Study risk factors such as advanced age, duration of disease, hypertension, smoking and hypercholesterolemia, but also SLE-related factors such as immune complex-induced endothelial injury, antiphospholipid antibody-induced thrombosis, complement activation, and corticosteroid exposure (3).

Accumulating evidence suggests that vascular inflammation and endothelial cell activation in lupus patients precedes CVD (8). Increased secretion of pro-inflammatory cytokines and chemokines by immune and non-immune cells, and binding of anti-dsDNA antibodies to cell surface cross-reactive antigens on resident renal cells can promote the loss of the protective endothelial glycocalyx and exposes cell adhesion molecules on endothelial cells, which in turn, promotes leukocyte recruitment. Shedding of glycocalyx constituents such as syndecan-1 (also known as CD138), hyaluronan (HA), thrombomodulin and cell adhesion molecules into the circulation may serve as soluble biomarkers of endothelial cell activation and dysfunction in LN. More recently, results from our group and others showed that circulating levels of these glycocalyx components are associated with disease activity in LN patients and may contribute to the pathogenesis of LN as well as CVD. It is pertinent to note that severe forms of LN, namely Class 3 or 4 in the ISN-RPS Classification (9, 10), are characterized by inflammatory cell infiltration in the glomerular capillaries, and it is not uncommon to observe endothelial cell abnormalities at the ultrastructural level under electron microscopy. As a result, abnormalities affecting vascular endothelial cells affect patients with LN at both microvascular and macrovascular levels. This review aims to give a brief overview of the endothelium, mechanisms of endothelial injury in LN, and recent advances in the understanding of endothelial glycocalyx constituents in the pathogenesis of LN, and their potential role as biomarkers for disease activity or complications in clinical management.

## 2 Endothelium

The vascular endothelium comprises a monolayer of endothelial cells that lines the entire circulatory system and separates the blood from the interstitial compartments. The integrity of the endothelium is dependent on intercellular junction complexes including tight junctions, adherens junctions and gap junctions; and integrins that facilitate the adhesion of endothelial cells to their underlying substratum (11). The endothelium plays critical roles in the regulation of vascular tone, permselectivity, platelet aggregation, blood coagulation, leukocyte trafficking, antigen presentation and lipoprotein catabolism (12, 13). Endothelial cells release numerous vasodilatory and vasoconstrictive molecules to maintain vascular homeostasis (11). Nitric oxide (NO) is an important vasoactive compound synthesized by endothelial cells through the conversion of L-arginine by nitric oxide synthase (NOS). NO maintains blood flow by regulating vasodilation, inhibits platelet aggregation and leukocyte adhesion, and prevents smooth muscle cell proliferation. The luminal surface of endothelial cells is lined by a protective glycocalyx which regulates many functions of blood vessels (**Table 1**), for example, thrombomodulin and heparan sulphate proteoglycans are key constituents of the glycocalyx and both exhibit antithrombotic actions.

**Table 1:** Functions of the glycocalyx.

FUNCTION	CONTRIBUTION OF THE GLYCOCALYX
**PERMSELECTIVITY BARRIER**	• Prevents circulating blood cells from permeating blood vessels • Filters small molecules • Restricts transvascular protein leakage • Prevents oedema by restricting passage of water and colloids through transendothelial and paracellular pathways • Regulates fluid shift and maintains fluid volume by acting as a negatively charged sieve
**VASCULAR TONE**	• Retains protective enzymes e.g. superoxide dismutase, which maintains NO bioavailability • Regulates eNOS • Regulates redox state
**MECHANORECEPTOR**	• Senses shear stress induced by viscous blood flow • ↑ fluid shear increases NO production → dilates blood vessels • High shear stress increases albumin uptake resulting in a thicker glycocalyx
**ANTI-COAGULATION**	• Maintains the antithrombotic property of endothelial cells
**CELL-TO-CELL COMMUNICATION**	• Preserves gap junctions through its attachment to the cytoskeleton of endothelial cells • Prevents platelet aggregation and thrombosis • Reduces endothelial cell contact with cellular and macromolecular components in the bloodstream • Provides a framework to bind plasma proteins • Prevents leukocyte-endothelial cell interaction

↑, increase; →, resulting in.

The endothelium also maintains the normal function of the kidney, a highly vascularized organ (14). The renal artery and its microvasculature provides blood to the kidney, delivers oxygen and nutrients to renal cells, and maintains the integrity of the endothelial monolayer through the expression of numerous proteins including interjunctional protein complexes, vascular endothelial cadherin, PECAM-1, VEGF/VEGFR complex, Tie2 and its ligands angiopoietin-1 (Ang1) and Ang2 (15). The microvasculature in the kidney cortex comprises afferent arterioles that direct blood into the glomerulus and efferent arterioles that transport blood from the glomerulus into peritubular capillaries surrounding tubules where they deliver blood into postcapillary venules. Renal endothelial cells are surrounded by support cells on their abluminal side, with smooth muscle cells supporting endothelial cells in arterioles and postcapillary venules, podocytes and mesangial cells supporting glomerular endothelial cells, and pericytes supporting peritubular capillaries (16). The microvasculature in the glomerulus serves as a selective permeability filter which permits small molecules such as water, sugars, electrolytes and small proteins to pass through while retaining high molecular weight proteins in the circulation (17). Peritubular microvessels remove waste molecules from the circulation into the urine and reabsorb molecules from pre-urine back into the blood. Compared to endothelial cells from other organs, fenestrated glomerular endothelial cells are endowed with a particularly thick glycocalyx that extends into the pores and also covers the surface of podocytes (18), and forms a critical component of the permeability barrier to solutes. Disturbances in the permeability of the glomerular capillary wall and loss of the glycocalyx contributes to albuminuria (19–21).

## 3 Endothelial glycocalyx

The luminal surface of endothelial cells is lined by a protective glycocalyx, also known as the pericellular matrix or endothelial surface layer. It is a delicate and heterogenous gel-like structure with a thickness ranging from 0.5 - 2 µm and it is composed of negatively-charged proteoglycans and glycosaminoglycans (GAGs) such as heparan sulphate, chondroitin sulphate and HA; glycoproteins bearing acidic oligosaccharides and terminal sialic acids; and plasma proteins including albumin and antithrombin. Glycoproteins such as selectins and cell adhesion molecules are located at the base of the glycocalyx and under physiological conditions, are obscured by a dense layer of proteoglycans covalently bound to the endothelial cell membrane. Proteoglycans such as syndecan-1, syndecan-4 and glypican-1, mask the binding sites of selectins and cell adhesion molecules thereby inhibiting leukocyte adhesion (22). On the luminal side, plasma proteins such as albumin are bound to GAG chains through electrostatic interactions between the negatively-charged sulphate and carboxylate groups on the GAG chains and positive arginine residues in albumin, and this interaction stabilizes the glycocalyx structure. The composition of the glycocalyx may vary depending on the blood vessel type, vascular bed and fluid shear stress applied to the endothelial cell surface (21). The glycocalyx synthesized by glomerular endothelial cells express more thrombomodulin compared to HUVEC but less than human brain microvascular endothelial cells, whereas expression of cell adhesion molecules are comparable between the aforementioned endothelial cells (23). The presence of proteoglycans and GAGs endows the glycocalyx with a high net negative charge that not only repels negatively charged molecules but prevents white blood cells and platelets from interacting with the endothelium. The glycocalyx serves to cushion and protect endothelial cells from injury and plays a vasculoprotective role by restricting transvascular protein leakage, leukocyte extravasation, thrombosis and complement activation; regulates redox state and NO release; and sequestration of antithrombotic factors, antioxidants, anti-inflammatory mediators, lipoproteins and protease inhibitors (24, 25) (**Table 2**). The glycocalyx also binds enzymes such as superoxide dismutase that protects the glycocalyx from oxidative stress. The integrity of the endothelial and glycocalyx barrier is regulated by endothelial stabilizing agents such as sphingosine-1-phosphate, which is transported to the endothelium by high density lipoprotein (HDL) and albumin, where it serves to stabilize the endothelial cortical actin cytoskeleton and inhibits secretion of matrix metalloproteinases (MMPs). These proteases degrade proteoglycans in the glycocalyx and matrix proteins which form the substratum on which endothelial cells adhere to (26).

**Table 2:** Constituents of the endothelial glycocalyx and their functions.

Glycocalyx constituents		Properties and Functions
**PROTEOGLYCAN:**	**Syndecan-1**	• Transmembrane proteoglycan containing heparan sulphate and chondroitin sulphate GAG chains • Binds chemokines and prevents them from interacting with their receptors • Serves as a cofactor for antithrombin • Release of heparan sulphate chains promotes inflammation
	**Glypican-1**	• Glycosylphosphatidylinositol anchored proteoglycan containing solely heparan sulphate GAG chains • Regulates endothelial cell inflammation, proliferation and endothelial cell-to-mesenchymal transition • Regulates arterial stiffness • Loss of glypican-1 is associated with decreased NO synthesis
**GAG**	**Hyaluronan**	• Preserves vascular integrity and homeostasis • Serves as a scaffold and microdomain for the binding and clustering of different receptors • Increased HA expression is associated with proteinuria • HA shedding and cleavage promotes inflammation
**GLYCOPROTEINS:**	**Selectins**	• E-selectin and P-selectin bind leukocytes through their glycosylated ligand and mediates their capture, resulting in their tethering and rolling on endothelial cells • Increased expression in inflammatory conditions • Increased in the glomerulus and interstitial microvasculature in lupus-prone mice
	**VCAM-1**	• Regulates tethering and firm adhesion of leukocytes and lymphocytes by binding to α4β1 integrin (VLA-4) and β2 integrin on immune cells • Weakly expressed in glomerular endothelial cells and proximal tubular epithelial cells in the normal kidney, increased expression in proliferative LN. • Expression is increased by cytokines, ROS, oxidized LDL • Release of VCAM-1 is associated with endothelial cell activation • VCAM-1 preserves its function in soluble form
	**ICAM-1**	• Regulates rolling, adhesion and crawling of leukocytes through its interaction with β2 integrin on leukocytes • Contributes to LN progression
	**PECAM-1**	• Regulates leukocyte extravasation
	**Integrins**	• Regulates endothelial cell and extracellular matrix interaction • Contribute to adhesion of leukocytes and platelets to the endothelium
	**Thrombomodulin**	• Possesses anticoagulant and anti-inflammatory properties • Inhibits blood coagulation • Binds thrombin and activates Protein C
**PLASMA PROTEINS:**	**Albumin**	• Binds to proteoglycans and contributes to the structural integrity and thickness of the glycocalyx • Transports sphingosine-1-phosphate, which when bound to its receptor, inhibits MMP activation and secretion, and prevents degradation of proteoglycans
	**Antithrombin**	• Binds to heparan sulphate and acts as a thrombin inhibitor

GAG, glycosaminoglycan; ICAM-1, intracellular cell adhesion molecule-1; MMP, matrix metalloproteinase; PECAM-1, platelet endothelial cell adhesion molecule-1; VCAM-1, vascular cell adhesion molecule-1.

Preserving the integrity of the glycocalyx is essential to maintain vascular homeostasis. Despite its important role in vasculoprotection, the glycocalyx is a fragile structure and can be easily damaged by changes in blood chemistry and flow patterns along the vessel walls, increased levels of hormones, neurotransmitters, vasoactive and atherogenic factors, TNF-α and MMPs, and a loss of plasma components especially albumin in the circulation (26–29). Inflammation or ischemia also induces endothelial cell activation, triggering downstream activation of proteases and shedding of the glycocalyx resulting in fluid extravasation, leukocyte and platelet adhesion, vascular inflammation, hypercoagulability, thrombosis, and loss of flow-responsive vasodilation (30). Shedding of the glycocalyx can reduce glycocalyx thickness by up to 65% and exacerbates systemic inflammatory processes by acting as danger associated molecular patterns (DAMPs) and initiate recruitment of neutrophils (30, 31). A loss of the glycocalyx leads to the acquisition of a non-adaptive phenotype characterized by a loss of the homeostatic mechanisms present in healthy endothelial cells. Alteration in the endothelial glycocalyx and composition is associated with CVD, hypertension and renal disease (19, 32–34) and precedes podocyte foot process effacement and proteinuria (35).

## 4 Endothelial injury in lupus nephritis

Endothelial cell activation, vascular inflammation and kidney injury can be induced through numerous effector mechanisms in LN and include the deposition of oxidized LDL (ox-LDL) in blood vessels, production of autoantibodies against endothelial cells and their subsequent binding to their cell surface antigens, induction of pro-inflammatory mediators including TNF-α, and formation of neutrophil extracellular traps (NETs).

Patients with Class 3 or 4 LN show glomerular endothelial injury, characterized by leukocyte accumulation and endocapillary proliferation associated with capillary wall destruction. These detrimental changes are initiated by immune complex deposition in the subendothelial space or thrombotic events in SLE-associated lupus anticoagulant syndrome (9, 10). The glomerular endothelium together with its negatively charged glycocalyx facilitates the deposition of positively charged autoantibodies (36). Deposition of immune complexes in the glomerular subendothelium or binding of anti-endothelial antibodies to cross-reactive antigens on the plasma membrane of endothelial cells results in the activation of procoagulation pathways, fibrin deposition and exudative lesions. Immune complex deposition, mediated in part through C5b-9 following complement activation, also increases secretion of cytokines and chemokines and expression of cell adhesion molecules leading to degradation of the glycocalyx, leukocyte recruitment, increased endothelial permeability, endothelial cell apoptosis and kidney injury (37–39). Inflammation is the body’s immediate response to tissue damage where circulating leukocytes adhere to activated endothelial cells and emigrate to the site of tissue damage with the aim of removing the injurious insult and start the healing process. This is a highly regulated, multi-step process comprising a series of coordinated interactions between leukocytes and endothelial cells. Neutrophils are the first-line responders to tissue injury and their adhesion to the vascular endothelium is essential to mount an inflammatory response. TNF-α released by macrophages at the site of injury facilitates the degradation of the endothelial glycocalyx constituents through activation of heparanase and MMP-9 in endothelial cells, and release of free radicals, proteases and heparanase from leukocytes and mast cells (40, 41). Shedding of heparan sulphate proteoglycans from the glycocalyx exposes selectins and cell adhesion molecules constitutively expressed on the endothelial cell surface, and engagement of P-selectin and E-selectin to sialylated, fucosylated mucin on leukocytes facilitate the initial attachment and rolling of leukocytes on the endothelium. Chemokines secreted by macrophages and stromal cells, which are immobilized by heparan sulphate proteoglycans in the glycocalyx, generate a chemotactic gradient that promotes activation of transmembrane G-protein coupled receptors on leukocytes, which initiates integrin clustering on leukocytes and their binding to VCAM-1, ICAM-1 and ICAM-2, leading to firm adhesion on the endothelium and subsequent extravasation or diapedesis (42–44). Secretion of pro-inflammatory cytokines such as IL-1, IFN-γ and TNF-α by tissue-resident macrophages and mast cells, and release of cytokines, chemokines and growth factors sequestered by heparan sulphate GAG chains following glycocalyx degradation, increases E-selectin, VCAM-1 and ICAM-1 expression and decreases thrombomodulin expression in endothelial cells, which augments neutrophil recruitment (23, 45). Increased expression of adhesion molecules on the luminal endothelial cell surface during inflammation is therefore associated with thinning of the glycocalyx. While glycocalyx degradation is crucial for neutrophilic response to danger signals, reconstitution of the glycocalyx is important for resolution of inflammatory processes. Elimination or suppression (as in the case of autoimmune diseases) of the injurious insult is essential to initiate both glycocalyx and tissue repair. In LN patients, a loss of self-tolerance, aberrant innate and adaptive immune responses and autoantibody production creates an inflammatory microenvironment in the kidney especially during relapse, and timely treatment is essential to prevent endothelial cell activation and vascular inflammation, and preserve patient and kidney survival (46).

Long-term use of immunosuppressive medication, impaired immune response, renal impairment and disease activity in LN patients is associated with an increased risk of infection (47). Neutrophils recruited to sites of infection release antimicrobial NETs, composed of chromatin and neutrophil components that trap and kill microbes in tissues and vasculature (48). Timely degradation and removal of NETs is crucial for tissue homeostasis and to prevent their presentation as autoantigens. DNase 1 is required to degrade NETs. LN patients have been reported to show impaired NET degradation consequent to increased serum level of DNase 1 inhibitors and production of anti-NET antibodies (49). Impairment of NET degradation correlates with disease activity in LN patients (49) and contribute to endothelial cell injury, vascular inflammation and thrombosis (48). Low density granulocytes (LDGs) are a distinct subset of pro-inflammatory neutrophils, which are increased in SLE patients and have been shown to promote endothelial cell injury in culture (50). Studies have shown that SLE-derived LGDs express high level of active MMP-9, which are externalized during NETosis and cluster on the plasma membrane of cultured endothelial cells and activates endothelial MMP-2 (51), which may contribute to shedding of the endothelial glycocalyx.

Chronic inflammation induces a plethora of inflammatory mediators including TNF-α, IL-1β and IL-6 and proteolytic enzymes, which augment shedding of endothelial glycocalyx constituents and their detection in the circulation and urine (52–56). Inflammation induces ROS and activation of MMPs, ADAMs and other proteases including elastase and cathepsin B, which induces cleavage of syndecan-1 and CD44 and releases their ectodomains from endothelial cells, while heparanase and hyaluronidase degrade heparan sulphate and HA GAG chains respectively. Although it was previously believed that MMPs were rapidly released following *de novo* synthesis, emerging evidence suggests that MMPs in their activated form are stored in secretory granules in the endothelial cells and can be rapidly released by inflammatory or angiogenic stimuli without prior upregulation of their mRNA (57). In experimental studies, considerable shedding of glycocalyx components in coronary vessels have been observed in isolated guinea pig hearts after 20 min following TNF-α perfusion (58), and this was associated with increased expression of cell adhesion molecules on the endothelium and reduced degradation of asymmetric dimethylarginine, the latter an endogenous NOS inhibitor (59). Serum TNF-α level is increase in LN patients with active disease and contribute to glycocalyx shedding and subsequent inflammatory processes in LN patients.

Endothelial injury leads to impaired vascular tone and permeability and reduced NO release resulting in diminished endothelium-dependent vasodilation and subsequent atherosclerotic lesion in SLE patients. Endothelial NOS (eNOS) expression inversely correlates with the degree of glomerular injury in LN patients, mediated in part through increased IFN-α signature (60, 61). Atherogenic risk and intimal-medial thickening have been reported to inversely correlate with the thickness of the glycocalyx (62). Aortic stiffness, defined as the reduced capability of an artery to expand and contract in response to changes in pressure (63), has been reported in SLE patients and predisposes patients to CVD. Artery stiffness associated with hypertension and ageing induces degradation of the endothelial glycocalyx (64, 65). HUVEC cultured on polyacrylamide gels with a substrate stiffness of 10 kPa, which mimicked subendothelial stiffness detected in aged or diseased arteries, showed a marked reduction in heparan sulphate GAG chains and glypican-1 core protein but not HA expression, and was accompanied by increased MCP-1 secretion and ICAM-1 expression when compared to cells cultured on gels with a subendothelial stiffness equivalent to that in healthy arteries (2.5 - 5.0 kPa) (66). To date, the clinico-association of glypican-1 with disease activity in SLE or LN patients has not been documented, and further studies are warranted to determine the association of glycocalyx-associated glypican-1 and vascular stiffness in LN patients.

Lipoprotein oxidation is an early event in atherogenesis resulting in the formation of various oxidation products that trigger local immune responses (67), and may play an important role in premature atherosclerosis in SLE patients. Increased levels of oxidized epitopes on LDL have been reported in SLE patients and is associated with coronary or peripheral arterial disease, carotid plaque, increased intima-medial thickness score, and kidney involvement (68). Whereas HDL possesses anti-oxidant properties and removes ROS from ox-LDL and inhibits the expression of adhesion molecules on endothelial cells, ox-LDL contributes to inflammatory processes through induction of inflammatory mediators and monocyte recruitment. In SLE patients, total HDL level is decreased whereas pro-inflammatory LDL level is increased (69). Increased inflammatory processes in atherogenesis results in the shedding of the glycocalyx constituents into the circulation leading to an impairment in its selective permeability barrier, which in turn, results in transendothelial leakage of atherogenic LDL at lesion-prone arterial sites (70). Areas of glycocalyx damage have been shown to correlate with ox-LDL cholesterol uptake. In apolipoprotein E-deficient mice, lipid accumulation and plaque formation increase in areas of the endothelium that showed reduced glycocalyx coverage, whereas plaque-free regions were observed in the endothelium with an intact glycocalyx (71, 72).

Endothelial cell apoptosis is a key feature of atherosclerosis and colocalizes with tissue factor activity in atherosclerotic plaques, possibly through activation of the redox-sensitive pathways and redistribution of phosphatidylserine residues (73, 74). Increased circulating apoptotic endothelial cells and decreased bone marrow-derived endothelial progenitor cells and myeloid angiogenic cells suggests an imbalance between endothelial cell injury and repair in SLE and LN patients (73). Stimulation of glomerular endothelial cells with serum from SLE and LN patients increases IL-1β and IL-6 transcripts and IL-8, IL-15 and PDGF-BB secretion and is associated with increased neutrophil recruitment (75, 76). These pro-inflammatory mediators also induce glycocalyx shedding.

Numerous physiological and pathological stimuli can alter endothelial permeability. Thrombin, histamine and pro-inflammatory cytokines can induce opening of adherens and tight junctional complexes through increased PKC phosphorylation, which in turn mediates cytoskeleton reorganization, altered expression of focal adhesion components including vinculin, α-actinin and talin, resulting in changes in cell-matrix interactions, a loss of cell-cell contact and increased paracellular transport (77). Although the PKC isoenzymes that mediated increased endothelial permeability was not determined, it is possible that PKC-α and PKC-β isoforms were increased since they are the predominant isoforms present in endothelial cells (78). We previously demonstrated that anti-dsDNA antibodies induced PKC-α, PKC-βI and PKC-βII phosphorylation in human mesangial cells and their expression were increased in the glomeruli in NZB/W F1 mice with active nephritis (79). Immune deposition in the subendothelial region is a characteristic feature in severe LN. It is possible that anti-dsDNA or other autoantibodies could bind to glomerular endothelial cells to induce endothelial injury through PKC activation or other signaling pathways. High molecular weight HA has been shown to reduce renal PKC activation in a murine model of diabetic nephropathy (80). Given that HA is a key component of the glycocalyx, its cleavage or shedding from the glycocalyx may induce PKC activation and subsequent acceleration of endothelial cell activation and dysfunction.

## 5 Clinical association of circulating glycocalyx constituents in patients with lupus nephritis

Early diagnosis of LN and monitoring of treatment efficacy is challenging, since conventional parameters used in clinical practice, such as proteinuria or deranged kidney function, are likely preceded by a stage of preclinical kidney injury. Kidney biopsy remains the gold standard in the diagnosis of LN and subsequent assessment of kidney histopathology but since it is invasive it is performed infrequently and cannot be repeated frequently. Conventional parameters for the assessment of kidney function such as proteinuria and serum creatinine are non-specific, and do not accurately reflect histopathological changes (54). Serial monitoring of anti-dsDNA and C3 levels is useful in some, but not all, patients (81, 82). There is currently no biomarker that shows a distinct advantage for flare prediction (83). Recently, we and others have demonstrated that measurement of glycocalyx constituents in serum and urine samples correlate with disease activity in LN.

Shedding of the glycocalyx constituents into the bloodstream is considered an early sign of endothelial activation and injury (34), and measurement of circulating glycocalyx components may serve as biomarkers of CVD and kidney injury in LN patients. Circulating fragments of the glycocalyx are emerging as diagnostic and prognostic tools for numerous pathological conditions including autoimmune diseases and sepsis. The next section will focus on circulating glycocalyx constituents that show association with disease parameters in SLE and LN patients.

### 5.1 Syndecan-1

Heparan sulphate comprises more than 50% of the total GAG content in the glycocalyx. It is synthesized in the Golgi apparatus and is composed of repeating disaccharide units of D-glucuronic acid and N-acetyl glucosamine residues. During biosynthesis, the GAG chain can be modified by N-deacetylation or N-sulphation of the glucosamine unit, O-sulphation of both monosaccharide units or C5 epimerization of glucuronic acid to iduronic acid (84). Given the different combination of modifications that can occur, the extent of sulphation and their location along the GAG chain, there is considerable structural diversity and 23 different disaccharides in heparan sulphate chains have so far been identified (85), which endow heparan sulphate GAG chains with the ability to bind to numerous ligands that mediate various biological functions (84). Heparan sulphate GAG chains are attached to a core protein and are present in the glycocalyx predominantly as syndecan-1.

Syndecan-1 is a transmembrane proteoglycan that has five potential attachment sites for GAG chains and may contain both heparan sulphate and chondroitin/dermatan sulphate GAG chains. Heparan sulphate GAG chains are restricted to the N terminus of the core protein whereas chondroitin/dermatan sulphate GAG chains are attached at the C terminus (86). Through its heparan sulphate GAG chains, syndecan-1 can interact with numerous extracellular matrix proteins such as collagen, laminin and fibronectin and it can serve as co-receptors for integrins and growth factors such as FGF, EGF and HGF, and inhibit IFN-γ and TNF-α activity (87, 88). Owing to these interactions, syndecan-1 plays key roles in cell proliferation, adhesion, signaling, wound healing and inflammation (88–90). Syndecan-1 can impede leukocyte adhesion to the endothelium by inhibiting the interaction of β2 integrin on leukocytes to ICAM-1 on endothelial cells. Transmigration of leukocytes from the circulation, across the endothelium and basement membrane to the site of injury is mediated through a chemotactic gradient. Syndecan-1 can immobilize chemokines such as CXCL1, 2 and 8 through electrostatic interactions between the negative charge of heparan sulphate GAG chains and the positive charge present in arginine and lysine residues in chemokines (85, 91), which alters the orientation and oligomerization of chemokines, which in turn, affects the interaction of chemokines with their receptors (91, 92). In patients with renal disease including LN, syndecan-1 present on tubular epithelial cells can bind L-selectin and MCP-1, and the degree of binding is associated with leukocyte infiltration (93). In addition to the glycocalyx, tubular epithelial cells may be another source of syndecan-1 in the kidney which can contribute to tubulo-interstitial fibrosis. In lupus-prone mice, syndecan-1 has been shown to interact with death receptor 6, an orphan immune regulator, which regulates expansion and activation of autoreactive follicular helper T cells and disease progression (94). A loss of syndecan-1 may exacerbate immunological responses in LN.

Endothelial cells are constantly exposed to the mechanical shearing forces of blood flow. Changes in the magnitude of shear stress in addition to temporal and spatial distribution have been reported to alter endothelial permeability and hydraulic conductivity and expression of cell adhesions. Fluid shear stress is a potent regulator of vascular homeostasis and induces AKT, Rho A and paxillin phosphorylation in endothelial cells, which play critical roles in NO production, regulation of oxidative stress and cell survival, cytoskeletal remodeling and adherence of endothelial cells to their underlying substratum (95–99). Heparan sulphate proteoglycans have been shown to act as mechanosensors (100). A loss of syndecan-1 in cultured endothelial cells through gene silencing inhibited AKT and Rho A activation and abolished the establishment of a paxillin phosphorylation gradient in response to flow, and induced a pro-atherosclerotic phenotype with decreased gene expression of Kruppel-like factor (KLF)-2 and KLF-4 and increased expression of KLF5 (101). KLF are a family of transcription factors and key regulators of vasomotor tone, inflammatory and thrombotic processes, and shear stress-induced phenotypic changes in endothelial cells. Whereas KLF-2 and KLF-4 can increase genes that are atheroprotective, KLF-5 induces gene expression of pro-inflammatory mediators in endothelial cells. These studies support a role for syndecan-1 in mechanosensing in cultured endothelial cells and suggests an atheroprotective role for syndecan-1 (101), and loss of syndecan-1 is associated with a pro-inflammatory phenotype. Pro-inflammatory mediators including TNF-α or IL-6 induces loss of syndecan-1 through increased MMP activation, which cleaves syndecan-1 ectodomain. Heparanase is an enzyme or sheddase that specifically cleaves heparan sulphate GAG chains from their protein core and is secreted by numerous cells including macrophages, podocytes and mast cells. Enzymatic removal of heparan sulphate GAG chains from the glycocalyx of cultured human glomerular endothelial cells resulted in an increase in albumin flux across the cell monolayer (102). Furthermore, increased glomerular heparanase expression is associated with proteinuria, increased BUN and glomerular injury (103, 104). The release of heparan sulphate GAG chains from syndecan-1 will in turn release a plethora of growth factors and cytokines sequestered on the GAG chains, triggering downstream inflammatory and fibrotic processes.

LN patients with active disease have increased serum syndecan-1 level compared to patients in remission and the level correlated with serological and clinical parameters of disease including anti-dsDNA antibody titre, proteinuria, serum creatinine level and both SLEDAI-2K and renal SLEDAI-2K scores (56, 105, 106). Proteinuria and renal SLEDAI-2K score are reliable markers of renal activity in LN and they reflect kidney injury. When compared to proteinuria and renal SLEDAI-2K score, serum syndecan-1 level showed comparable correlation with serological markers of active disease, and a better correlation with serum creatinine level and eGFR. Serum syndecan-1 level was also higher in LN patients with active disease compared to SLE patients with extrarenal manifestations (53, 56) and this may be attributed, at least in part, to a loss of syndecan-1 from the glomerular endothelial glycocalyx and downstream glomerular injury. Serum syndecan-1 level showed a higher sensitivity rate (85.91%) than anti-dsDNA antibody titre (75.00%) and C3 level (62.07%) in distinguishing patients with active LN from quiescent LN patients, while the specificity rate was comparable between all three markers (86.21%, 91.67% and 96.43% respectively) (53). Although anti-dsDNA antibody and C3 levels reflect serological activation, not all episodes of relapse are preceded by an increase in these clinical indicators. Serum syndecan-1 level also correlated with the severity of interstitial inflammation in renal biopsies from LN patients with active disease (53, 56). One possible mechanism through which soluble syndecan-1 contributes to interstitial inflammation may be through the release of chemokines after syndecan-1 shedding from tubular epithelial cells, which facilitates ligand-receptor interaction and recruitment of mononuclear cells (107). Serum syndecan-1 level has also been reported to correlate with circulating CD20^-^CD38^+^CD138^+^ plasma cells suggesting that syndecan-1 may also be derived from CD138^+^ plasma cells (105). Soluble syndecan-1 can activate B cell differentiation and autoantibody production in a murine model of SLE (108), whereas heparan sulphate fragments can induce secretion of pro-inflammatory cytokines and low molecular weight HA which exacerbate inflammatory processes through TLR-4 and highlights a pathogenic role of syndecan-1 in SLE and LN (109, 110). In a longitudinal study, increased serum syndecan-1 level preceded clinical flare by 3 to 4 months and decreased after treatment in parallel with clinical improvement suggesting that measurement of serum syndecan-1 level may be useful in monitoring impending disease flare (53). Whether syndecan-1 level might be related to cardiovascular complications in SLE patients remains to be investigated but it may be a predictor of endothelial cell activation. Despite an increase in serum syndecan-1 level, none of the LN patients in the longitudinal study developed CVD at the time of recruitment (53). Since endothelial cells play a key role in the early events of atherosclerosis, it is not surprising that endothelial cell activation and injury is observed in asymptomatic patients. Increased plasma syndecan-1 level and decreased capillary glycocalyx thickness is observed in patients with antiphospholipid syndrome and may contribute to endothelial injury and vascular thrombosis that are characteristic features in this condition (111). Increase plasma syndecan-1 level may serve as an independent risk factor for adverse cardiovascular events in patients with non-ischemic dilated cardiomyopathy (112).

### 5.2 Hyaluronan

HA is a non-sulphated GAG composed of repeating disaccharide units of D-glucuronic acid and N-acetyl-D-glucosamine and is synthesized on the inner surface of the plasma membrane by one of three isoforms of HA synthase (HAS), namely HAS-1, HAS-2 and HAS-3. Nascent HA molecules are extruded through the plasma membrane onto the cell surface or into the extracellular matrix. Despite its simple chemical structure, HA has pleiotropic functions that depend on its molecular weight and tissue distribution. High molecular weight HA possesses anti-inflammatory and anti-angiogenic properties, whereas low molecular weight HA generated by either *de novo* synthesis or degradation of the parent molecule by ROS or hyaluronidase, possesses pro-inflammatory and angiogenic properties, and may exacerbate tissue inflammation, through its ability to induce chemokine secretion in macrophages (113). Its anionic properties are attributed to the presence of carboxyl groups. Unlike other GAGs, HA is not covalently attached to a core protein and is bound and tethered to endothelial cells by binding to CD44, its principal cell surface receptor. At the cell surface, HA polymerization generates macromolecules with molecular weight in excess of 1,000 kDa, with a length ranging from 2 - 25 µm. HA accounts for 5 - 20% of the total GAG content in the glycocalyx, and HA cables intertwines in the glycocalyx and functions as a scaffold and microdomain for the binding and clustering of different receptors and contributes to the permselectivity of vessels by repelling or preventing the passage of plasma proteins, thereby creating a small protein-free zone in the more compact inner layer of the glycocalyx. HA also contributes to the oncotic gradient directed towards the lumen of the blood vessel, which limits water efflux out of the bloodstream, and this gradient is reduced following the loss of HA from the glycocalyx. In animal studies, inactivation of HAS-2 in glomerular endothelial cells decreased HA expression in the endothelial glycocalyx by 80%, and this was associated with vascular destabilization characterized by capillary ballooning, loss of endothelial fenestrations, mesangiolysis, glomerulosclerosis and development of albuminuria (114). A loss of HA also resulted in a reduction of Ang1/Tie2 signaling, the latter a key regulator of endothelial quiescence and maintenance of endothelial cell barrier (114).

In a murine model of atherosclerosis, pharmacological inhibition of HA synthesis using 4-methylumbelliferone reduced the thickness of the endothelial glycocalyx and this was accompanied by endothelial dysfunction, increased thrombotic and inflammatory responses with macrophage retention in vascular lesions and a more rapid progression to atherosclerosis (115). Exposure of the glycocalyx to hyaluronidase also resulted in a marked decrease in glycocalyx thickness with a concomitant increase in capillary wall permeability and pericapillary oedema (116). In diabetic patients, the extent of glycocalyx shedding and release of HA into the circulation correlates with carotid artery intimal-medial thickness (117). A similar correlation may exist in LN patients although further studies are warranted to confirm this. A loss of glycocalyx HA, whether by enzymatic cleavage or pharmacological inhibition, promotes atherosclerotic lesion and highlights the importance of glycocalyx HA in maintaining homeostatic vascular function. Proteolytic degradation of CD44 by cathepsin B, elastase or thrombin can also release HA from the glycocalyx and may exert similar detrimental effects (118).

In the vasculature, HA is incorporated into the glycocalyx or extracellular matrix. Although glycocalyx HA plays a key role in preserving vascular integrity and homeostasis, recent studies have shown that it also contributes to progressive LN by acting as a ligand for CD44^+^ T cell binding (119). In healthy subjects, circulating HA has a half-life of 2 - 5 min and is rapidly removal from the circulation by the liver and kidney (120). Serum HA level is increased in LN patients with active disease compared to patients in remission, which may be attributed to either impaired clearance or increased synthesis, and it correlated with serological and clinical parameters of disease (53, 121). Whereas serum syndecan-1 level increased prior to clinical flare, HA level increase at the time of nephritic flare, decreased after treatment and returned to basal level after 9 months (53). Serum HA level showed similar sensitivity but lower specificity than anti-dsDNA antibody or C3 levels (sensitivity and specificity rates: 74.07% and 68.96% respectively for HA, 75.00% and 91.67% respectively for anti-dsDNA antibodies, and 62.07% and 96.43% respectively for C3), and lower sensitivity and specificity rates than proteinuria (sensitivity and specificity rates of 85.71% and 87.50% respectively) and renal SLEDAI-2K scores (sensitivity and specificity rates of 100.00% and 100.00% respectively) in distinguishing LN patients with active disease and remission (53). HA level was comparable in patients with LN and non-renal SLE, and given that HA plays an important role in inflammatory disorders and is expressed by both immune and non-immune cells (122), it is not surprising that HA level cannot distinguish between active LN and active non-renal SLE patients. Serum HA level correlates with tubular atrophy score, interstitial fibrosis score and overall chronicity index and showed no association with activity index or its components suggesting that HA may contribute to chronic kidney disease (53). In CKD patients, plasma syndecan-1 level was detected in patients with stage 4 CKD, whereas increased HA level was observed only in patients with stage 5 CKD suggesting that shedding of HA occurs later than that of syndecan-1 and may be indicative of more severe injury to the kidney (123).

HA has been reported to drive inflammatory and fibrotic processes in the lungs through activation of CD44 and TLR-4 (122). HA also contributes to kidney fibrosis in lupus-prone mice and this may explain at least in part, the association of HA with histologic chronicity index. HA expression is increased in the glomerulus of LN patients, mediated through anti-dsDNA antibody induction of HAS-2 and IL-1β and downstream synthesis of both high and low molecular weight HA (121). Low molecular weight HA may be generated by HAS-3 activation or through enzymatic degradation of high molecular weight HA by hyaluronidases. Given that anti-dsDNA antibodies had no effect on HAS-3 activation (121), an increase in low molecular weight HA may be due to enzymatic cleavage of the glomerular endothelial glycocalyx. Low molecular weight HA exacerbates inflammatory processes through its ability to expose cell adhesion molecules in the glycocalyx and subsequent macrophage binding and recruitment.

LN is characterized by aberrant influx of immune cells into the kidney. T cell homing in the glomerulus is mediated through the binding of CD44 on T cells to HA in the endothelial glycocalyx. The glycocalyx is thus the first point of contact between circulating immune cells and the local microenvironment and may contributes to pathogenesis of disease (119). In contrast to other experimental models of kidney disease where a reduction of endothelial glycocalyx is observed, murine models of LN have shown a 3-fold increase in the thickness of the endothelial glycocalyx, attributed to increased HA expression and this was accompanied by proteinuria, whereas removal of HA from the endothelial glycocalyx using hyaluronidase removed the number of activated T cells in the glomerulus and improved proteinuria (119). Whether an increase in glomerular endothelial glycocalyx thickness is also observed in LN patients has not been investigated. We and others have demonstrated that glomerular HA expression is increased in LN patients, mediated in part through anti-dsDNA antibodies and increased HAS-2 expression (121, 124). It is possible that HA plays a protective and pathogenic role in LN depending on its molecular weight.

### 5.3 Thrombomodulin

Thrombomodulin is a 74 kDa transmembrane protein that is predominantly expressed in the endothelial glycocalyx and to a lesser extent on mesangial cells, mesothelial cells, dendritic cells and monocytes (125, 126). Thrombomodulin acts as a membrane-bound, high-affinity receptor for thrombin and prevents its interaction with platelets and coagulation factors. Following its binding to thrombin, thrombomodulin activates protein C, which in turn degrades coagulation factors (127–129). Thrombomodulin may also contain chondroitin sulphate GAG chains which may serve as a weak ligand for thrombin (130). Thrombomodulin generates an anti-inflammatory and barrier-stabilizing microenvironment and has been shown to regulate NFκB signaling, IL-6 secretion and expression of cell adhesion molecules including ELAM-1, VCAM-1 and ICAM-1 (131). Decreased thrombomodulin expression in the endothelial glycocalyx is attributed to reduced transcription and translation induced by pro-inflammatory cytokines such as TNF-α (132), or through cleavage by neutrophil proteinases, with a concomitant increase in soluble thrombomodulin in serum, plasma and urine in patients with renal disease including LN (53, 133–135).

Increased soluble thrombomodulin level is associated with endothelial dysfunction and vascular risk, atherosclerosis, cardioembolic stroke and obesity (136). We and others have reported that serum thrombomodulin level is increased in patients with active LN compared to LN patients in remission, patients with non-renal SLE, CKD patients and healthy subjects (53, 133–135, 137–141). Serum thrombomodulin level correlated with serological and clinical parameters of disease including anti-dsDNA antibody level, renal SLEDAI-2K score, proteinuria and serum creatinine level, and inversely correlated with eGFR, serum albumin and C3 levels (53, 56, 135). In a longitudinal study, increased serum thrombomodulin level preceded clinical flare by almost 4 months and as with syndecan-1, may be a potential indicator of impending disease flare (53). Unlike syndecan-1 and HA levels which returned to baseline levels after approximately 9 months of treatment, serum thrombomodulin level persisted for a longer duration (53). Whether this suggests abnormality of endothelial cell function, ongoing low-grade immune-mediated inflammation or progressive kidney damage warrants further investigation. Serum thrombomodulin level can distinguish between patients with active LN and healthy subjects and patients with active non-renal SLE but did not show a high specificity in distinguishing LN patient and CKD patients suggesting that increased thrombomodulin level may be a potential biomarker of kidney damage (53). In a Multi-Ethnic Study of Atherosclerosis (MESA), serum thrombomodulin level showed a strong inverse correlation with glomerular filtration rate (142). When compared to conventional serological markers of active LN, thrombomodulin level was more sensitive (89.66%) than anti-dsDNA antibody and C3 levels in distinguishing active LN from remission and showed less specificity (68.97%) to conventional biomarkers (53). Serum thrombomodulin level correlated with the severity of interstitial inflammation in renal biopsies from patients with active LN and may reflect histopathological changes, for example, renal vascular lesions in LN is associated with increased plasma thrombomodulin levels (53, 134), and patients with end-stage kidney disease show a noticeable loss of their glycocalyx in the sublingual microvasculature, which is accompanied by an increase in plasma thrombomodulin levels compared with healthy subjects (125). Kidney transplantation replenishes the endothelial glycocalyx and can reduce serum thrombomodulin levels, except in patients who developed allograft CKD when thrombomodulin level remained elevated (125).

### 5.4 Cell adhesion molecules

Adhesion of circulating leukocytes to the endothelium and their transmigration to the site of injury is mediated through cell adhesion molecules such as E-selectin, VCAM-1 (also known as CD106) and ICAM-1. Under non-inflammatory conditions, the glycocalyx serves as the first line of defense against leukocyte adhesion and limits the interaction of leukocytes with cell adhesion molecules. Under inflammatory conditions, shedding of the endothelial glycocalyx exposes cell adhesion molecules, which permits circulating leukocytes to adhere to the endothelium followed by diapedesis. Diseases characterized by acute inflammation are often associated with increased E-selectin expression, whereas chronic inflammation is associated with increased expression of VCAM-1 and ICAM-1 (143). VCAM-1 and ICAM-1 expression is increased during systemic and local inflammation by TNF-α and IL-1β, and they initiate the strong adhesion of circulating leukocytes to the endothelium and their subsequent transmigration through endothelial cell junctions (144).

Adhesion molecules are shed from the activated endothelium following proteolytic cleavage and are detected in the circulation where they could potentially serve as soluble biomarkers of vascular endothelial dysfunction and cardiovascular disease (145, 146). Plasma VCAM-1 and E-selectin are associated with cardiovascular events, coronary calcium, and carotid plaques in SLE patients (147, 148). VCAM-1 and ICAM-1 expression have also been detected in the glomeruli and renal tubules in patients and mice with active LN, suggesting that resident renal cells may also contribute to circulating levels of adhesion molecules (149, 150).

Early studies that investigated serum VCAM-1 level in SLE and Class 3 or 4 LN patients were conflicting (133, 151–153). Independent researchers have shown an association between serum VCAM-1 level and proteinuria, but not with anti-dsDNA antibody or C3 levels, or SLEDAI score (134), while other studies demonstrated an increase in VCAM-1 level in SLE patients but no association with disease activity or organ involvement (154). Correlation between serum VCAM-1 level and coronary calcification/subclinical atherosclerosis has also been reported, irrespective of whether the patient had lupus (155). We recently demonstrated that serum VCAM-1 level was significantly higher in LN patients with nephritic flare compared to remission, and its level correlated with clinical and serological parameters of disease including anti-dsDNA antibody level, renal SLEDAI score and proteinuria and inversely correlated with C3 level. Serum VCAM-1 level also showed high sensitivity and specificity and could distinguish between patients with active LN from those in remission (sensitivity and specificity rates of 68.97% and 89.66% respectively), patients with active non-renal SLE (sensitivity and specificity rates of 90.91% and 86.21% respectively), CKD patients (sensitivity and specificity rates of 89.66% and 82.61% respectively) and healthy subjects (96.55% and 96.00% respectively) (52). When compared to conventional biomarkers of disease, serum VCAM-1 level showed comparable sensitivity and specificity as anti-dsDNA antibody and C3 levels in distinguishing patients with active LN and remission, and similar sensitivity and specificity as C3 (sensitivity and specificity rates of 90.00% and 89.29% respectively), but higher specificity than anti-dsDNA antibody titre (sensitivity and specificity rates of 100.00% and 42.86% respectively), in distinguishing active LN patients from patients with active non-renal SLE. The measurement of a panel of biomarkers rather than a single biomarker, may be more useful to predict nephritis flares (156, 157) and VCAM-1 in combination with C3, proteinuria or serum levels of syndecan-1, HA and thrombomodulin was superior to C3, anti-dsDNA antibody titre or serum creatinine level in distinguishing active LN from quiescent disease (52). In a longitudinal study, serum VCAM-1 level increased 4.5 months before nephritic flare was evident clinically as shown by an increase of serum creatinine level and/or proteinuria and VCAM-1 level returned to baseline after one year, suggesting that serum VCAM-1 level may serve as an early indicator of impending nephritic flare. Serum VCAM-1 level correlated with serum levels of syndecan-1, HA and thrombomodulin suggesting a close association between components of the endothelial glycocalyx. At the time of flare, serum VCAM-1 level correlates with leukocyte infiltration score and fibrinoid necrosis/karyorrhexis score in renal biopsies from LN patients highlighting its role in leukocyte infiltration into the kidney (52). Increased urine VCAM-1 level has also been reported in patients with active LN and strongly correlated with renal biopsy activity score. In this respect, urine VCAM-1 level has been proposed to serve as a noninvasive marker in assessing histopathologic changes in the kidney and is associated with inferior long-term renal outcome (54, 158–161). In childhood-onset SLE, urinary VCAM-1 outperformed anti-dsDNA antibody titre and C3 level as biomarkers to predict nephritic flare (55). Using aptamer-based screening followed by ELISA validation, urinary VCAM-1 was identified as a strong candidate to distinguish between active LN and quiescent disease irrespective of ethnicity (162). More recently, the assessment of urine: serum fractional excretion ratios of cell adhesion molecules outperformed corresponding urine and serum levels in identifying active LN from remission (163).

As with VCAM-1, conflicting results have also been reported for serum ICAM-1 and E-selectin levels in SLE and LN patients and their association with clinical and serological parameters of disease (52, 152, 162, 164–171). The discrepancies may be related to differences in the characteristics of patients, ethnicity and management, the timing of sample collection in relation to disease activity, and differences in the sensitivity of assays used (52, 134, 172). In our study, whereas VCAM-1 showed a high seropositivity rate (93%) in patients with active LN, a lower seropositivity rate was observed for ICAM-1 at 38%. Although the longitudinal profile of ICAM-1 followed a similar trend to that of disease activity and response to treatment, serum ICAM-1 level showed no association with anti-dsDNA antibody or C3 levels. In addition to its expression on endothelial cells, VCAM-1 and ICAM-1 expression has also been observed on monocytes, mesangial cells, proximal tubular epithelial cells and smooth muscle cells. Increased VCAM-1 expression is observed in the kidney of lupus-prone mice with active disease when compared to non-autoimmune mice, suggesting a role in mediating leukocyte infiltration in the inflamed kidney parenchyma (149). The mechanisms though which renal VCAM-1 or ICAM-1 expression is induced in LN remain to be fully defined although their induction by anti-dsDNA antibodies has been proposed (173, 174).

## 6 Conclusions

Early diagnosis of LN, accurate assessment of disease activity and timely monitoring of treatment efficacy is essential for patient and kidney survival, but this remains challenging due to the heterogeneity and complexity of LN. A renal biopsy remains the gold standard for the diagnosis and prognosis of LN but since it is invasive, it is not feasible to perform frequent kidney biopsies for routine monitoring of histopathologic changes and disease activity. Conventional clinical and serological parameters to assess disease activity rely on non-specific indicators of kidney injury or function such as proteinuria and serum creatinine level, which could present late in the course of disease activation and do not reflect histopathologic changes. Serological tests for anti-dsDNA antibody and C3 levels reflect immunological status but not kidney injury and not all patients show an association of anti-dsDNA antibody titre with disease activity. Identification of novel biomarkers for LN should facilitate early diagnosis of nephritic flare and monitoring of treatment response in order to preserve residual kidney function. Biomarkers for LN should accurately reflect disease activity, be reliable and useful in clinical practice, easily measured routinely, have biological and pathophysiological relevance across ethnicities, and show superiority to current conventional markers (175). Furthermore, biomarkers for LN should be able to distinguish between LN and non-renal SLE patients. Given the heterogeneity and complexity of disease, it is unlikely that one molecule can serve as a biomarker for LN. Over the past few decades, biomarker discovery in LN has progressed from individual candidates to unbiased high-throughput platforms mass spectrometry, proteomics and aptamer-based screening. Urinary biomarkers have yielded promising results in predicting the activity of LN, but their clinical utility requires further assessment. The current challenge in the development of novel biomarkers following their discovery for LN is their validation in a large population with ethnic diversity. A biomarker panel may provide superior information regarding LN compared to a single marker, without overlap with other types of glomerulonephritis (176).

**Figure 1:**
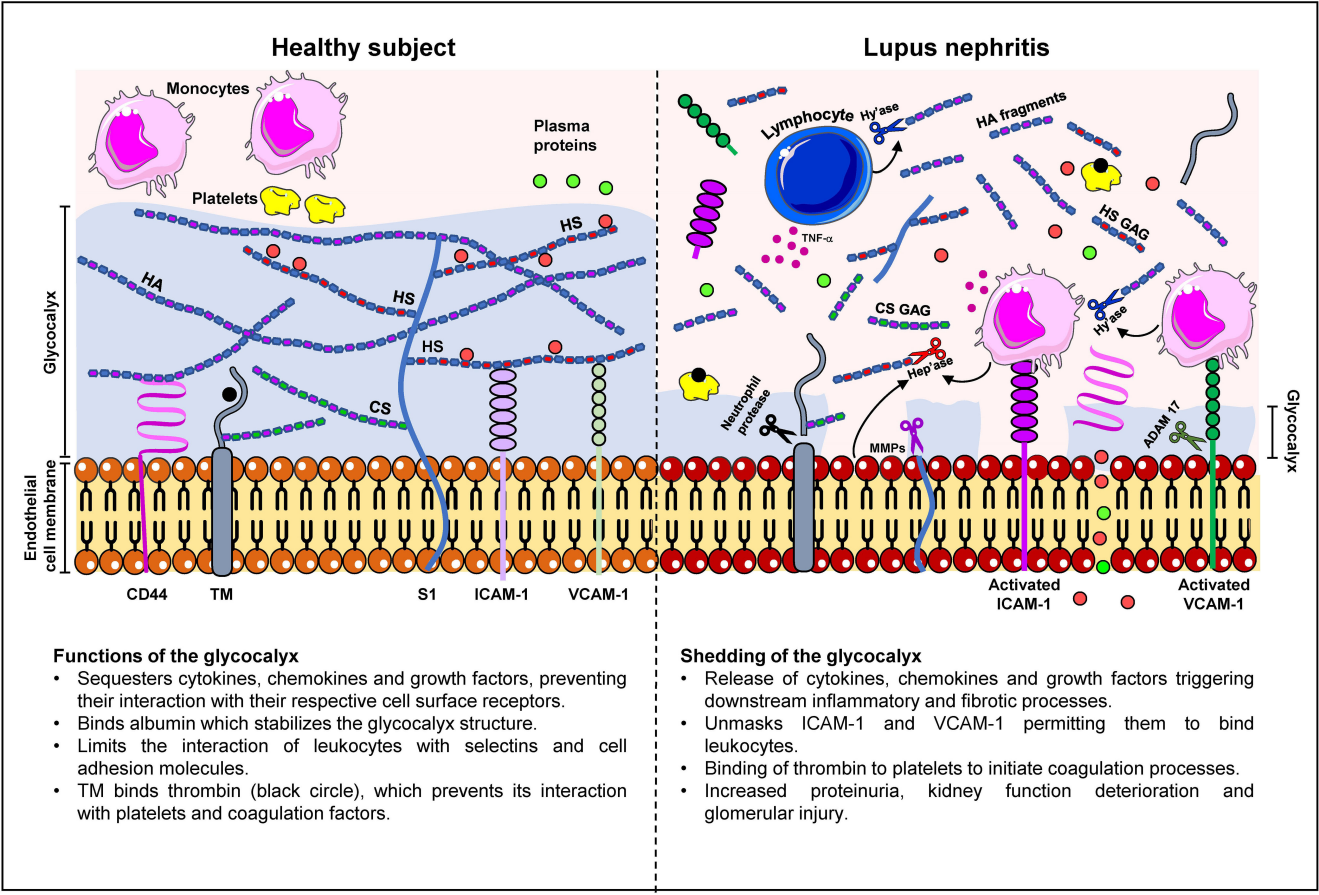
Changes in the glycocalyx composition in lupus nephritis patients. In a healthy subject, the glycocalyx ranges from 0.5 - 2.0 μm in thickness and comprises negatively-charged proteoglycans, glycosaminoglycans (GAG) and glycoproteins such as syndecan-1 (S1), CD44, HA, thrombomodulin (TM), and cell adhesion molecules. The glycocalyx serves to cushion and protect endothelial cells and restrict transvascular protein leakage. The interaction of albumin (red circle) with heparan sulphate GAG chains (HS GAG) stabilizes the glycocalyx structure. S1 and TM may also contain chondroitin sulphate (CS) GAG chains which contribute to the electronegative charge of the glycocalyx. The high net negative charge of the glycocalyx prevents leukocytes and platelets from interacting with the endothelium and contributes to the maintenance of endothelial permeability to plasma proteins (green circles). Proteoglycans also mask the binding sites of selectins and cell adhesion molecules, which inhibits leukocyte adhesion. In LN, chronic inflammation is accompanied by increased cytokine, chemokine and growth factor secretion in immune and non-immune cells, which in turn, increases synthesis of enzymes that cleave glycocalyx constituents leading to the destruction of the glycocalyx. These enzymes include hyaluronidase (Hy’ase) that cleaves HA, heparanase (Hep’ase) that cleaves HS GAG chains, MMPs that cleave syndecan-1 ectodomain and CD44, neutrophil protease that cleaves TM, and ADAM-17 that cleaves cell adhesion molecules. Shedding of the glycocalyx releases pro-inflammatory mediators that were bound to glycocalyx constituents and unmasks the binding sites in VCAM-1 and ICAM-1 thereby permitting the binding of leukocytes and exacerbation of inflammation. Shedding of the glycocalyx also contributes to transvascular protein leakage and proteinuria.

We and others have shown that constituents of the glycocalyx correlate with disease activity and may have potential diagnostic and prognostic value in LN, endothelial cell activation and possible cardiovascular complications. **Table 3** compares the clinico-pathological association of serum syndecan-1, HA, thrombomodulin and VCAM-1 with conventional indicators of kidney injury and immunologic activity in LN patients. Further studies are warranted in a larger cohort to validate these findings especially in longitudinal studies and different ethnic groups to ascertain whether certain biomarker panels may be used for specific ethnicity and genetic composition. A schematic diagram detailing changes in the glycocalyx in LN patients is presented in **Figure 1**.

**Table 3:** Comparison of syndecan-1, hyaluronan, thrombomodulin and VCAM-1 as putative biomarkers for lupus nephritis compared to conventional serological and clinical markers of disease.

	Syndecan-1	HA	TM	VCAM-1	Anti-dsDNA	C3	Proteinuria	Renal SLEDAI-2K
**active LN and healthy subjects:**	96% sensitivity 96% specificity	74% sensitivity 88% specificity	100% sensitivity 100% specificity	96% sensitivity 96% specificity	ND	ND	ND	100% sensitivity 100% specificity
**active LN and** **remission:**	85% sensitivity 86% specificity	74% sensitivity 68% specificity	89% sensitivity 68% specificity	68% sensitivity 89% specificity	75% sensitivity 91% specificity	62% sensitivity 96% specificity	85% sensitivity 87% specificity	100% sensitivity 100% specificity
**active LN and active non-renal lupus:**	100% sensitivity 70% specificity	63% sensitivity 74% specificity	90% sensitivity 100% specificity	90% sensitivity 86% specificity	100% sensitivity 42% specificity	90%sensitivity 89% specificity	100% sensitivity 100% specificity	100% sensitivity 100% specificity
Correlates with:								
**Proteinuria**	Yes	Yes	Yes	Yes	Yes	Yes	–	Yes
**Serum creatinine level**	Yes	Yes	Yes	Yes	Yes	No	No	No
**Renal SLEDAI**	Yes	Yes	Yes	Yes	Yes	Yes (inverse)	Yes	–
**SLEDAI**	Yes	Yes	Yes	Yes	Yes	Yes (inverse)	Yes	Yes
**Anti-dsDNA antibody level**	Yes	Yes	Yes	Yes	–	Yes	Yes	Yes
**Components of histologic activity indices:**	Yes (interstitial inflammation score)	No	Yes (interstitial inflammation score)	Yes (leukocyte infiltration score)	No	No	No	No
**Histologic chronicity score:**	No	Yes	No	No	No	No	No	No
Inverse correlation with:								
**eGFR**	Yes	No	Yes	No	No	No	No	No
**serum albumin level**	Yes	Yes	Yes	ND	Yes	Positive correlation	Yes	Yes
**C3 level**	Yes	Yes	Yes	Yes	Yes	–	Yes	Yes
**Detected before nephritic flare:**	Yes	No	Yes	Yes	Yes	No	No	No
**Serial monitoring of disease activity:**	Yes	Yes	Yes	Yes	Yes	Yes	Yes	Yes

C3, complement 3; HA, hyaluronan; TM, thrombomodulin; ND, not determined. Data obtained from (52, 53).

## Author contributions

Drafting the article: SY and TC. Approval of the final version for submission: SY and TC. All authors contributed to the article.

## Glossary

**Table:**

ACR	albumin-to-creatinine ratio
ADAM	a disintegrin and metalloproteinases
Ang	angiopoietin
AUC	area under curve
C3	complement 3
CD	cluster of differentiation
CKD	chronic kidney disease
CVD	cardiovascular disease
CXCL1	C-X-C motif chemokine ligand
DAMPs	danger associated molecular patterns
dsDNA	double-stranded deoxyribonucleic acid
EGF	epidermal growth factor
eGFR	estimated glomerular filtration rate
ELAM-1	epithelial cell adhesion moleculae-1
ELISA	enzyme-linked immunosorbent assay
eNOS	endothelial nitric oxide synthase
FGF	fibroblast growth factor
GAG	glycosaminoglycan
HA	hyaluronan
HDL	high-density lipoprotein
HGF	hepatocyte growth factor
ICAM-1	intercellular adhesion molecule-1
IFN	interferon
IL	interleukin
ISN-RPS	International Society of Nephrology/Renal Pathology Society
KLF	Kruppel-like factor
LDL	low-density lipoprotein
LN	lupus nephritis
MCP-1	monocyte chemoattractant protein-1
MMP	matrix metalloproteinase
NET	neutrophil extracellular traps
NFκB	nuclear factor kappa-light-chain-enhancer of activated B cells
NO	nitric oxide
NOS	nitric oxide synthase
PDGF	platelet derived growth factor
PECAM-1	platelet endothelial cell adhesion molecule-1
PG	proteoglycan
PKC	protein kinase C
ROC	receiver operating characteristic
ROS	reactive oxygen species
SLE	systemic lupus erythematosus
SLEDAI-2K	systemic lupus erythematosus disease activity index-2000
TM	thrombomodulin
TNF-α	tumor necrosis factor-α
VCAM-1	vascular cell adhesion molecule-1
VEGF	vascular endothelial growth factor
VEGFR	vascular endothelial growth factor receptor.
